# Antitumor necrosis factor treatment in patients with inflammatory bowel disease does not promote psoriasis development: A meta-analysis

**DOI:** 10.1097/MD.0000000000029872

**Published:** 2022-07-08

**Authors:** Yu Kyung Jun, Joo Young Park, Seong-Joon Koh, Hyunsun Park, Hyoun Woo Kang, Jong Pil Im, Joo Sung Kim

**Affiliations:** a Division of Gastroenterology and Hepatology, Department of Internal Medicine, Seoul National University Bundang Hospital, Seongnam, Republic of Korea; b Seoul National University College of Medicine, Seoul, Republic of Korea; c Division of Gastroenterology and Hepatology, Department of Internal Medicine, Seoul National University Hospital, Seoul, Republic of Korea; d Department of Dermatology, SMG-SNU Boramae Medical Center, Seoul, Republic of Korea; e Division of Gastroenterology and Hepatology, Department of Internal Medicine, SMG-SNU Boramae Medical Center, Seoul, Republic of Korea.

**Keywords:** anti-tumor necrosis factor, colitis, Crohn’s disease, inflammatory bowel disease, psoriasis, ulcerative

## Abstract

**Background:**

Recent case reports have suggested that anti-tumor necrosis factor (TNF) agents are associated with an increased risk of developing psoriasis in patients with inflammatory bowel disease (IBD).

**Aims:**

This meta-analysis of published studies aimed to evaluate the association between anti-TNF treatment and psoriasis in patients with IBD.

**Methods:**

An electronic search for original articles published before April 7, 2022, was performed using PubMed, EMBASE, and the Cochrane Library. Independent reviewers conducted the article screening and data extraction. Psoriasis development between anti-TNF-treated and anti-TNF-naïve patients was compared. Patients with ulcerative colitis and Crohn disease were compared with determine the differences in anti-TNF-induced psoriasis. Also, psoriasis development was compared according to the types of anti-TNF agents. Random-effects model meta-analyses, network meta-analysis, funnel plot asymmetry, Begg rank correlation test, and Egger regression test were performed to generate summary estimates and explore the possibility of publication bias.

**Results:**

We analyzed a total of 10,778 articles searched and 14 articles were selected to analyze. There was no significant difference in psoriasis development between anti-TNF-treated and anti-TNF-naïve patients (relative risk = 1.14; 95% confidence interval = 0.77-1.68). No differences were found for psoriasis development between anti-TNF-treated ulcerative colitis and Crohn disease patients (relative risk = 1.30; 95% confidence interval = 0.87-1.95). No significant difference was reported with respect to psoriasis development according to the types of anti-TNF agents. We found no definitive publication bias in our analyses.

**Conclusions:**

Anti-TNF treatment did not contribute to the psoriasis development in patients with IBD. Based on our study, anti-TNF agents may be used for IBD treatment without concern for psoriasis development.

## 1. Introduction

Inflammatory bowel disease (IBD) is a chronic inflammatory disease of the gastrointestinal tract that is typically identified as Crohn disease (CD) and ulcerative colitis (UC). The disease course of IBD is highly variable, and IBD treatment involves either medical therapy or surgery. Antitumor necrosis factor (TNF) agents, including infliximab, adalimumab, golimumab, and certolizumab, are effective for induction and maintenance of remission in patients with IBD and reduce the risk of surgery and mortality.^[1,2]^ However, long-term usage of anti-TNF agents is associated with complications, such as infection, postoperative wound healing failure, malignancy, and immune-mediated diseases.^[3,4]^ Psoriasis, one of the immune-mediated diseases, can occur in IBD patients with anti-TNF agents, but ironically anti-TNF agents are also effective to control psoriasis.

Psoriasis and IBD are genetically and pathologically similar. Similar genetic susceptibility loci have been observed for both psoriasis and IBD, although each susceptibility loci is often correlated with different genes. In a genome-wide association analysis, psoriasis and CD shared the same 7 susceptibility loci outside of the human leukocyte antigen region. Four of these were identified as psoriasis and CD risk loci, including *IL23R* and *IL12B* genes, which play an important role in the proinflammatory reaction of T helper (Th) 17 cells. Psoriasis and IBD also share similar pathogenesis. Defensive barriers, skin, and intestinal mucosa, lose their integrity during the development and progression of psoriasis and IBD. Th17 cells were key cells in the adaptive immune responses of both diseases.^[5]^ Th17 cells produce cytokines, such as interleukin (IL)-17 and IL-22. IL-17 levels are increased in the inflamed intestinal mucosa and skin of IBD and psoriasis patients, respectively. Because of genetic and pathogenic similarities, CD is a risk factor for concomitant psoriasis.

Patients with psoriasis are at an increased risk of developing IBD compared with the general population. Inflammatory markers and clinical severity scores are higher in psoriasis patients with concomitant IBD than in those without IBD.^[6]^ Furthermore, patients with IBD have a higher potential for developing concomitant psoriasis than those without IBD.^[7]^ Additionally, CD patients with concomitant psoriasis had a poorer disease course than those with CD only.^[8]^

Despite the close relationship between psoriasis and IBD, there has been little data on the paradoxical development of psoriasis after anti-TNF treatment in IBD patients. In this study, we aimed to determine the association between anti-TNF treatment and psoriasis development in patients with IBD by conducting a meta-analysis of published studies.

## 2. Methods

### 2.1. Data sources and search strategy

We searched PubMed, EMBASE, and Cochrane Library databases for published articles from their inception to April 7, 2022, using the search terms “inflammatory bowel disease,” “Crohn’s disease,” “ulcerative colitis,” and “psoriasis.” The detailed search strategy is described in Text 1, Supplemental Digital Content, http://links.lww.com/MD/G857.

### 2.2. Study selection

Two of the authors (Y.K.J. and J.Y.P.) independently assessed the eligibility of all studies based on predetermined selection criteria. Studies that met the following eligibility criteria were included: (1) clinical studies, such as cohort or case-control studies; (2) studies that evaluated the occurrence of psoriasis after anti-TNF therapy in patients with IBD; (3) studies in which the number of patients required for our meta-analysis was described; and (4) studies written in English. The exclusion criteria were as follows: (1) nonhuman studies; (2) reviews, editorials, expert opinions, letters, or case reports; (3) studies in which did not deal with anti-TNF-treated patients with IBD; (4) studies in which the order of psoriasis occurrence and the initiation of anti-TNF treatment was unclear; and (5) studies with severe heterogeneity. We included the most recent and thorough articles when duplicate publications were identified. Disagreements between the evaluators were resolved through discussion.

The authors evaluated each study according to the Preferred Reporting Items for Systematic Review and Meta-Analysis Protocols (PRISMA) guidelines, which were revised in 2020. Investigators made the systematic process of analysis and review of articles. Three investigators (S.J.K., Y.K.J., and J.Y.P.) reviewed the selected articles individually. Discrepancies were discussed and resolved. All of the selected articles were case-control or cohort studies. Therefore, the Newcastle-Ottawa Scale was used to judge the quality of the included articles. In accordance with the Newcastle-Ottawa Scale system, the authors assessed the quality of the selected articles without bias (Table 1, Supplemental Digital Content, http://links.lww.com/MD/G857). This study did not qualify for ethics approval because it is a meta-analysis based on published articles.

### 2.3. Statistical analysis

All statistical analyses were performed using R statistical software (version 4.1.4; R Project for Statistical Computing, Vienna, Austria). Relative risk (RR) and 95% confidence intervals (CIs) were calculated using a random-effects model. This study used RR because a greater number of cohort studies than case-control studies were included. For estimating heterogeneity across trials, we used the $I^{2}$ statistical test. An $I^{2}$ value ≥50% was considered to indicate substantial heterogeneity. We performed a frequentist random-effect network meta-analysis using the graph theory approach. Inconsistencies were assessed by global and local inconsistency tests and if there were no significances in both tests (*P* ≥ .5), the consistency model was accepted. Publication bias was evaluated by the funnel plot asymmetry, Begg rank correlation test, and Egger regression test. A *P* value <0.5 indicated significant publication bias.

## 3. Results

### 3.1. Literature search

**Figure 1.**
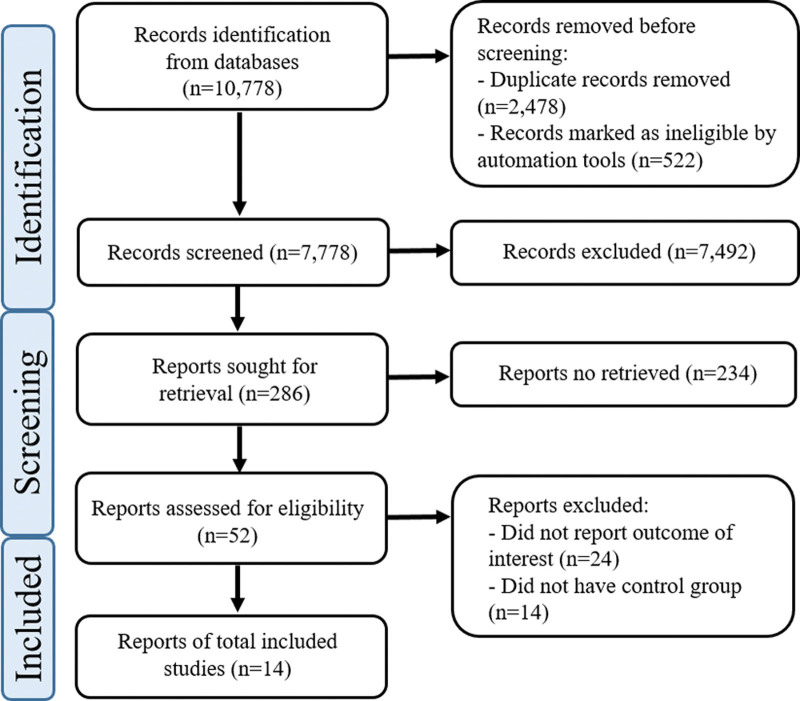
Flow diagram of study selection. CD = Crohn’s disease; n = number; TNF = tumor necrosis factor; UC = ulcerative colitis.

We identified 10,778 potentially eligible articles through the database searches, and 2478 duplicated articles were excluded. Two hundred eighty-six articles were selected after evaluation of the study title, 52 articles were retrieved after examining the abstract of these articles, and 14 articles were selected after full-text assessment. First, 7 articles were selected to assess the difference in psoriasis development between IBD patients who were treated or not treated with anti-TNF agents. Ten articles were selected to evaluate the difference in psoriasis development between CD and UC patients who had anti-TNF therapy. And 7 articles were used to compare the psoriasis development according to the types of anti-TNF agents. Figure 1 summarizes the literature search and selection process.

### 3.2. Study characteristics and quality

The baseline characteristics and quality assessment of the 14 articles are presented in Table 1.^[9–22]^ The majority of the selected studies were cohort studies; however, 3 nationwide population studies based on claims data and 3 case-control studies were included. Among anti-TNF agents, infliximab was used more frequently, followed by adalimumab. A total of 51,412 IBD patients were analyzed using the data from 14 articles.

**Table 1:** Main characteristics of all studies used in the meta-analysis (ordered by publication year and author)

Author	Publication, yr	Country	Study design	Data source	Study duration (yr)	Male (%)	IBD patients	CD patients	UC patients	Mean age at time of IBD diagnosis (yr)	Anti-TNF agents	NOS
Guerra et al^[9]^	2012	Spain	Multicenter retrospective cohort study	EMR review			1294	1087	207		Infliximab, adalimumab	6
Afzali et al^[10]^	2014	USA	Single-center retrospective cohort study	EMR review		29.6	27	26	1	39.7 ± 10.5	Infliximab, adalimumab, certolizumab	5
George et al^[11]^	2015	USA	Single-center case-control study	EMR review	2004–2013		94	73	21		Infliximab, adalimumab, certolizumab	5
Lolli et al^[12]^	2015	Italy	Single-center case-control prospective study	Dermatological assessment	2011–2013	54	251	158	93		Infliximab, adalimumab, certolizumab	6
Guerra et al^[13]^	2016	Spain	Nationwide population study	Spanish ENEIDA registry data	2015*		7415†	5488	1822		Infliximab, adalimumab, golimumab, certolizumab	7
Protic et al^[14]^	2016	Switzerland	Single-center case-control study	Dermatological assessment	2010–2013		752‡	239	458		Infliximab, adalimumab, certolizumab	6
Kirthi et al^[15]^	2017	Italy	Single-center retrospective cohort study	EMR review	2000–2015	51	1384	901	483		Infliximab, adalimumab	7
Vavricka et al^[16]^	2017	Switzerland	Nationwide cohort study	Questionnaire data	2006–2010		1249				Infliximab, adalimumab, certolizumab	7
Andrade et al^[17]^	2018	Portugal	Single-center observational study	Outpatient EMR review	2005–2015	48	732	610	122	29 ± 12	Infliximab, adalimumab	6
Bae et al^[18]^	2018	Korea	Nationwide population study	Korean NHIC data	2007–2016	67	16,284	11,751	4533		Infliximab, adalimumab	7
Weizman et al^[19]^	2018	Canada	Single-center retrospective cohort study	EMR review	2004–2016	49	676	497	165	21 ± 10.2	Infliximab, adalimumab	7
Courbette et al^[20]^	2019	France	Single-center retrospective cohort study	EMR review	2002–2014	51	147	123	24	14 ± 2.2	Infliximab	7
Burckley et al^[21]^	2021	USA	Single-center observational study	EMR review	2008–2018		3035				Infliximab, adalimumab, etanercept, certolizumab, golimumab	6
Ward et al^[22]^	2021	Denmark	Nationwide population study	Danish National Patient Register cohort	2005–2018	47.2	18,072	8811	9261		Infliximab, adalimumab, golimumab	7

If there was no relevant information, the column was left blank.

CD = Crohn disease, EMR = electronic medical record, IBD = inflammatory bowel disease, NHIC = National Health Insurance Claim, NOS = Newcastle-Ottawa scale, TNF = tumor necrosis factor, UC = ulcerative colitis.

* Guerra et al’s study included patients in the ENEIDA registry until March 2015.

† One hundred five patients were classified as IBD unclassified among the 7415 patients.

‡ Fifty-five patients were classified as IBD unclassified among the 752 patients.

### 3.3. The effect of anti-TNF therapy on psoriasis development in IBD patients

**Figure 2.**
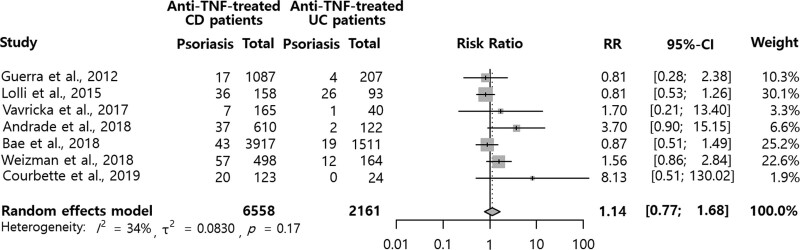
Psoriasis in inflammatory bowel disease patients with or without anti-TNF agents. CI = confidence interval; RR = relative risks; TNF = tumor necrosis factor.

First, we identified the occurrence of psoriasis in patients with IBD after anti-TNF treatment in 7 studies. A total of 8719 patients with IBD, composed of 6558 anti-TNF-treated and 2161 anti-TNF-naïve patients, were included in the study population. As shown in Figure 2, psoriasis development was not significantly associated with anti-TNF treatment in IBD patients (RR = 1.14, 95% CI = 0.77-1.68). This analysis had considerable heterogeneity ($I^{2}$= 34%). Significant publication bias was not discovered in the funnel plot (Figure 1, Supplemental Digital Content, http://links.lww.com/MD/G857), Begg rank correlation test (*P* = .8510), and Egger regression test (*P* = .7218).

### 3.4. The difference in psoriasis risk between CD and UC patients after anti-TNF therapy

**Figure 3.**
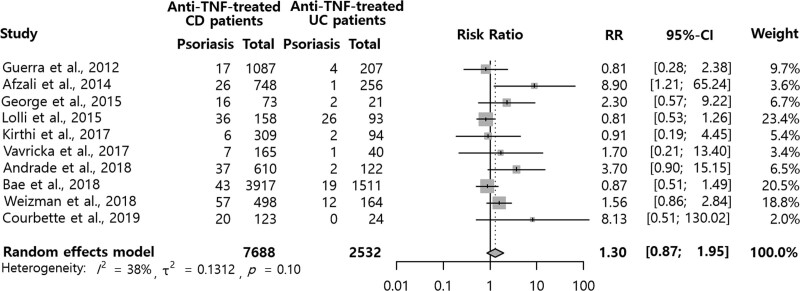
Psoriasis in patients with Crohn disease or ulcerative colitis after anti-TNF treatment. CD = Crohn disease; CI = confidence interval; RR = relative risks; TNF = tumor necrosis factor; UC = ulcerative colitis.

A total of 10,220 IBD patients treated with anti-TNF agents, composed of 7688 CD and 2532 UC patients, were included in the study population. The meta-analysis results for the difference in psoriasis risk between CD and UC patients after anti-TNF therapy are shown in Figure 3. Post-anti-TNF psoriasis risk was not significantly different between CD and UC patients (RR = 1.30, 95% CI = 0.87-1.95; the higher the RR, the higher the likelihood of developing psoriasis in CD patients than in UC patients). The heterogeneity of this analysis was not significant ($I^{2}$= 38%). The funnel plot showed slight asymmetry (Figure 2, Supplemental Digital Content, http://links.lww.com/MD/G857). However, there was no significant publication bias observed in the Begg rank correlation test (*P* = .8510) or the Egger regression test (*P* = .7218).

### 3.5. The risk of psoriasis according to the kinds of anti-TNF agents in IBD patients

**Figure 4.**
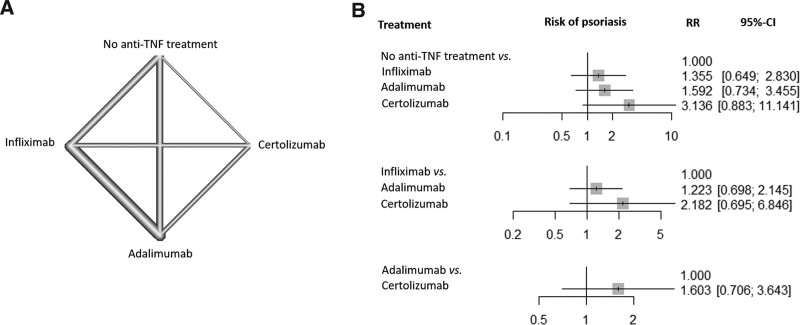
(A) Network plot of binary outcomes for psoriasis risk in patients with inflammatory bowel disease. (B) The binary comparisons of psoriasis risk in patients with inflammatory bowel disease according to the types of anti-TNF agents. CI = confidence interval; RR = relative risks; TNF = tumor necrosis factor.

Seven studies provided information about the kinds of anti-TNF agents for IBD treatment. According to the kinds of anti-TNF agents, 4 subgroups were identified and analyzed: No anti-TNF treatment, infliximab, adalimumab, and certolizumab treatments. The network plot of network analysis is shown in Figure 4. The thickness of lines was weighted according to the number of studies including anti-TNF agents treatment or comparison. All anti-TNF treatments did not increase the risks of psoriasis development relative to treatment without anti-TNF agents. Inconsistencies were assessed with global and local inconsistency tests, and both results reported no significant inconsistency. There was no significant publication bias observed in the Begg rank correlation test (*P* = .7187) or the Egger regression test (*P* = .6457).

## 4. Discussion

The use of anti-TNF agents has been rapidly increasing over the last 20 years in patients with IBD. However, the association between the use of anti-TNF agents and the development of psoriasis in patients with IBD remains unclear. Our study found that the psoriasis development did not increase after anti-TNF therapy in patients with IBD. There was no difference in anti-TNF-induced psoriasis risk between CD and UC patients, unlike previous studies reporting that CD was a risk factor for psoriasis. Additionally, the types of anti-TNF agents did not influence psoriasis development in IBD patients. Therefore, we believe that this study might be the first to comprehensively examine the association between anti-TNF therapy and paradoxical psoriasis development in patients with IBD.

The incidence of psoriasis in the general population varies from 30.3 per 100,000 person-years to 321.1 per 100,000 person-years according to age and country. However, psoriasis development in IBD patients is reported to be higher than that in the general population. The incidence of psoriasis was 217.68 per 100,000 person-years and 228.62 per 100,000 person-years for UC and CD, respectively. Patients with IBD are more likely to develop psoriasis than the non-IBD population regardless of the use of anti-TNF agents. However, in the current meta-analysis, post-anti TNF psoriasis in anti-TNF-treated IBD patients was not higher than that in anti-TNF-naïve counterparts. Our study demonstrated that anti-TNF agents were not associated with the risk of developing psoriasis. Based on these findings, we believe that IBD itself is the most important risk factor for developing psoriasis.

Both psoriasis and IBD are associated with T-cell activation and production of proinflammatory cytokines, such as IL-17A, IL-23, and TNF-α, but biologics targeting IL-17A, IL-23, or TNF-α showed different therapeutic effects in psoriasis or IBD patients. Anti-TNF agents have shown therapeutic efficacy in both IBD and psoriasis. The IL-23/IL-17A pathway is a major target for psoriasis treatment and anti-IL-23/IL-17A agents are superior to anti-TNF agents in psoriasis.^[23]^ Ustekinumab, binding to the P40 subunit of IL-12 and IL-23, has been approved for use in both diseases.^[24]^ Risankizumab and mirikizumab, selective anti-IL-23 agents, were highly effective in psoriasis, and clinical trials are ongoing to evaluate the efficacy and safety of the anti-IL-23 agents in patients with IBD. However, the availability of anti-IL-17A agents for IBD treatment was debatable because of IBD development in patients treated with secukinumab and ixekizumab.^[25]^

The current study might be the first meta-analysis of the comparison of psoriasis development in anti-TNF-treated and anti-TNF-naïve patients with IBD, although there have been many studies supporting a positive relationship between psoriasis and IBD. The strength of our study is that it comprehensively demonstrated that anti-TNF treatment did not influence the paradoxical development of psoriasis and the types of anti-TNF agents were not related to the risk of psoriasis in IBD patients. Articles eligible for meta-analysis were systematically selected. Most of the selected studies were qualified cohort studies, and publication bias was not significant. In addition, the difference between CD and UC in psoriasis development after anti-TNF treatment was also assessed. Our results showed that patients with CD had a similar risk of paradoxical psoriasis compared with UC patients, which was inconsistent with previous studies.

The current study has some limitations. The number of included articles was not large, and the heterogeneity of analyses was considerable. Some studies contained missing information on adult patients and only included children. Of the 14, 9 studies were conducted in Europe and 4 studies in the United States, with a relatively small proportion of Asians. The disease severity of included IBD patients was unreliable, and several studies only included patients who were serious enough to require anti-TNF treatments. Information on psoriasis development described in the articles was obtained from various sources, including electronic medical records and dermatologic assessments, patient questionnaires, and health insurance claim databases, and these data sources had different reliabilities. Moreover, detailed information on the duration of anti-TNF treatment, the time gap between the initiation of anti-TNF treatment and psoriasis development, dose of anti-TNF agents, and combination therapy with immunosuppressants was not available.

In conclusion, we found no evidence that anti-TNF agents increase the risk of psoriasis development in patients with IBD. The risk of psoriasis in anti-TNF-treated patients was not different according to either IBD types (UC or CD) or anti-TNF agent types (infliximab, adalimumab, or certolizumab). Our study can help physicians develop a treatment strategy for IBD patients.

## Author contributions

Conceptualization and Methodology: YKJ, SJK, HSP. Formal analysis: YKJ, JYP. Project administration: YKJ, SJK, HSP. Writing-original draft: YKJ. Writing-review and editing: YKJ, SJK, HSP. Approval of final manuscript: all authors.

## Supplementary Material


