# Supplementary material for: Antitumor necrosis factor treatment in patients with inflammatory bowel disease does not promote psoriasis development: A meta-analysis
Source: Medicine (Baltimore). 2022 Jul 8;101(27):e29872. doi: 10.1097/MD.0000000000029872 (PMC9259149; doi:10.1097/MD.0000000000029872)
Supplement: Supplementary file 1 [file medi-101-e29872-s001.pdf]

S1 Text. Supplementary Methods.

Search strategies for PubMed, EMBASE, and the Cochrane Library

PubMed

(((((((((((((((((((((((((Inflammatory Bowel Diseases[MeSH Terms]) OR Inflammatory Bowel Disease*[Title/Abstract]) OR Crohn's Enterit*[Title/Abstract]) OR Regional Enterit*[Title/Abstract]) OR Crohn's Disease*[Title/Abstract]) OR Crohns Disease*[Title/Abstract]) OR Crohn Disease*[Title/Abstract]) OR Granulomatous Enterit*[Title/Abstract]) OR Ileocolit*[Title/Abstract]) OR Granulomatous Colit*[Title/Abstract]) OR Ulcerative Colit*[Title/Abstract]) OR Colitis Grav*[Title/Abstract]) OR pancolit*[Title/Abstract]) OR Proctocolitis[MeSH Terms]) OR Hemorrhagic Rectocolit*[Title/Abstract]) OR Hemorrhagic Proctocolit*[Title/Abstract]) OR Ulcerative Rectocolit*[Title/Abstract]) OR Ulcerative Proctocolit*[Title/Abstract]) OR Proctosigmoidit*[Title/Abstract]) OR ulcerative proctosigmoidit*[Title/Abstract])) AND ((((((Psoriasis[MeSH Terms]) OR Pustulosis Palmaris et Plantaris[Title/Abstract]) OR Palmoplantaris Pustulosis[Title/Abstract]) OR (Pustular Psoriasis of Palms[Title/Abstract] AND Soles[Title/Abstract])) OR psoriatic palmoplantaris pustulosis[Title/Abstract]))

EMBASE

#1: 'inflammatory bowel disease'/exp OR 'inflammatory bowel disease*':ab,ti OR 'crohn* enterit*':ab,ti OR 'regional enterit*':ab,ti OR 'crohn* disease*':ab,ti OR 'granulomatous enterit*':ab,ti OR ileocolit*:ab,ti OR 'granulomatous colit*':ab,ti OR 'ulcerative colit*':ab,ti OR 'colitis grav*':ab,ti OR pancolit*:ab,ti OR 'proctocolitis'/exp OR proctocolit*:ab,ti OR 'hemorrhagic proctocolit*':ab,ti OR 'hemorrhagic rectocolit*':ab,ti OR 'ulcerative rectocolit*':ab,ti OR 'ulcerative proctocolit*':ab,ti OR proctosigmoidit*:ab,ti OR rectosigmoidit*:ab,ti OR 'ulcerative proctosigmoidit*':ab,ti

#2: 'psoriasis'/exp OR psorias*:ab,ti OR 'pustulosis palmaris et plantaris':ab,ti OR 'palmoplantaris pustulosis':ab,ti OR ('pustular psoriasis of palms and soles':ab,ti) OR 'psoriatic palmoplantaris pustulosis':ab,ti

#1 AND #2

Cochrane Library

#1	MeSH descriptor: [Inflammatory Bowel Diseases] explode all trees
#2	(Inflammatory Bowel Disease*):ti,ab,kw
#3	(Crohn's Enterit*):ti,ab,kw OR (Crohn's Disease*):ti,ab,kw OR (Crohns Disease*):ti,ab,kw OR (Crohn Disease*):ti,ab,kw
#4	(Granulomatous Enterit*):ti,ab,kw OR (Ileocolit*):ti,ab,kw OR (Granulomatous Colit*):ti,ab,kw
#5	(Idiopathic Proctocolit*):ti,ab,kw OR (Ulcerative Colit*):ti,ab,kw OR (Colitis Grav*):ti,ab,kw OR (pancolit*):ti,ab,kw
#6	MeSH descriptor: [Proctocolitis] explode all trees
#7	(Rectocolit*):ti,ab,kw OR (Hemorrhagic Rectocolit*):ti,ab,kw OR (Hemorrhagic Proctocolit*):ti,ab,kw OR (Ulcerative Rectocolit*):ti,ab,kw OR (Ulcerative Proctocolit*):ti,ab,kw
#8	(Proctosigmoidit*):ti,ab,kw OR (Rectosigmoidit*):ti,ab,kw OR (ulcerative proctosigmoidit*):ti,ab,kw
#9	#1 OR #2 OR #3 OR #4 OR #5 OR #6 OR #7 OR #8
#10	MeSH descriptor: [Psoriasis] explode all trees
#11	(Psorias*):ti,ab,kw OR (Pustulosis Palmaris et Plantaris):ti,ab,kw OR (Palmoplantaris Pustulosis):ti,ab,kw OR (Pustular Psoriasis of Palms and Soles):ti,ab,kw OR (psoriatic palmoplantaris pustulosis):ti,ab,kw
#12	#10 OR #11
#13	#9 AND #12

S1 Table.

Quality assessment of included case-control studies by the Newcastle-Ottawa scale

	**Selection**				**Comparability**	**Exposure**		
	Is the case definition adequate?	Representativeness of the cases	Selection of controls	Definition of controls	Comparability of cases and cohorts on the basis of the design or analysis	Ascertainment of exposure	Same method of ascertainment for cases and controls	Non-Response rate
George et al., 2015		★-		★	★	★	★	
Lolli *et al.,* 2015		★-		★	★	★	★	★
Protic *et al.,* 2016	★	★-		★	★	★	★	

Quality assessment of included cohort studies by the Newcastle-Ottawa scale

	**Selection**				**Comparability**	**Outcome**		
	Representativeness of the exposed cohort	Selection of the non-exposed cohort	Ascertainment of exposure	Demonstration that outcome of interest was not present at start of study	Comparability of cohorts on the basis of the design or analysis	Assessment of outcome	Was follow-up long enough for outcomes to occur	Adequacy of follow-up of cohorts
Guerra *et al.,* 2012	★	★	★		★	★	★	
Afzali et al., 2013	★		★		★	★	★	★
Guerra *et al.,* 2015	★	★	★		★	★	★	★
Kirthi *et al.,* 2017	★	★	★		★	★	★	★

Vavricka *et al.,* 2017	★	★	★		★	★	★	★
Andrade *et al.,* 2018	★	★	★		★	★	★	
Bae *et al.,* 2018	★	★	★	★	★	★	★	
Weizman *et al.*, 2018	★	★	★		★	★	★	★
Courbette *et al.,* 2019	★	★	★	★		★	★	★
Burckley *et al.,* 2021	★	★	★		★	★	★	
Ward et al., 2021	★	★	★		★	★	★	★

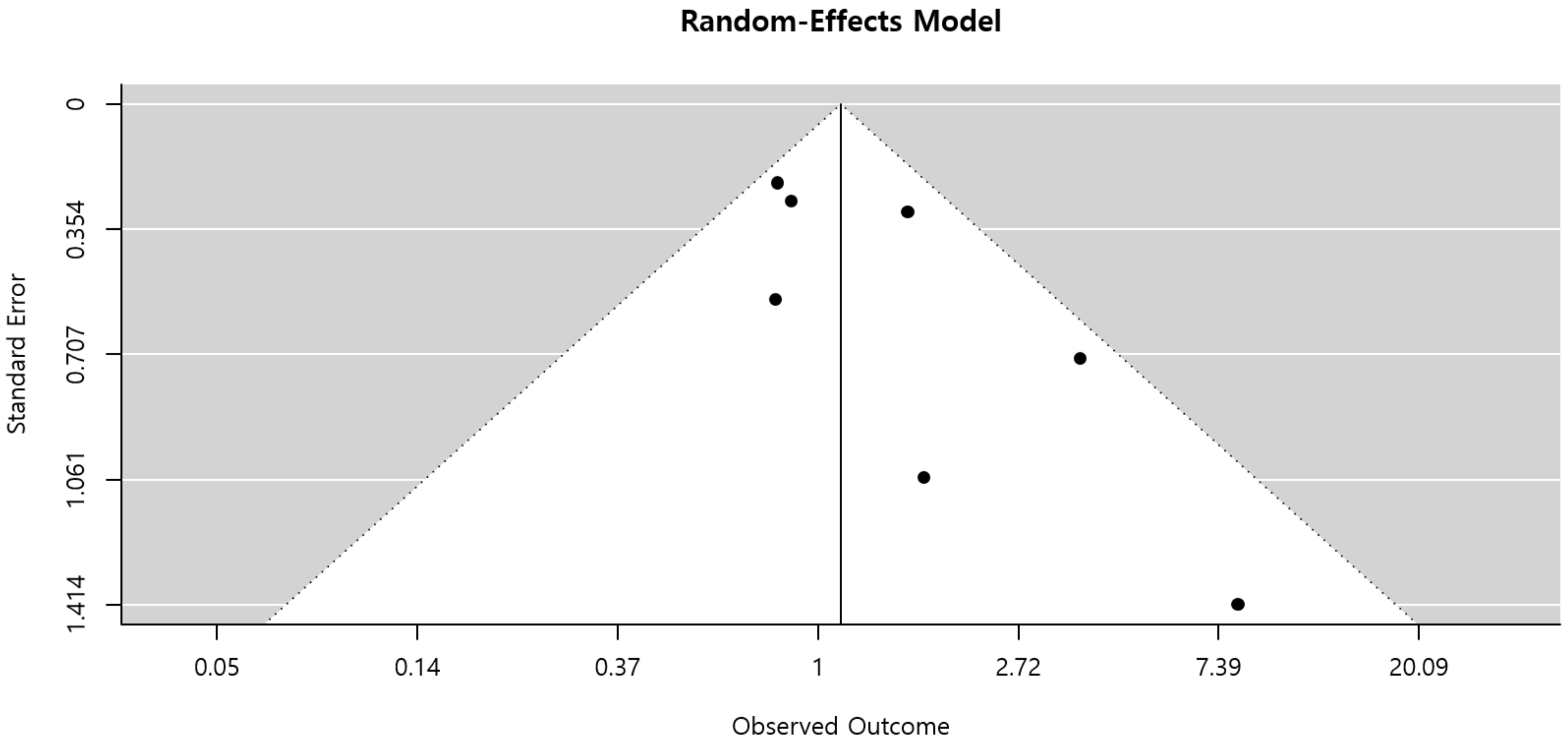
Random-Effects Model
Standard Error
0
0.354
0.707
1.061
1.414
0.05
0.14
0.37
1
2.72
7.39
20.09
Observed Outcome

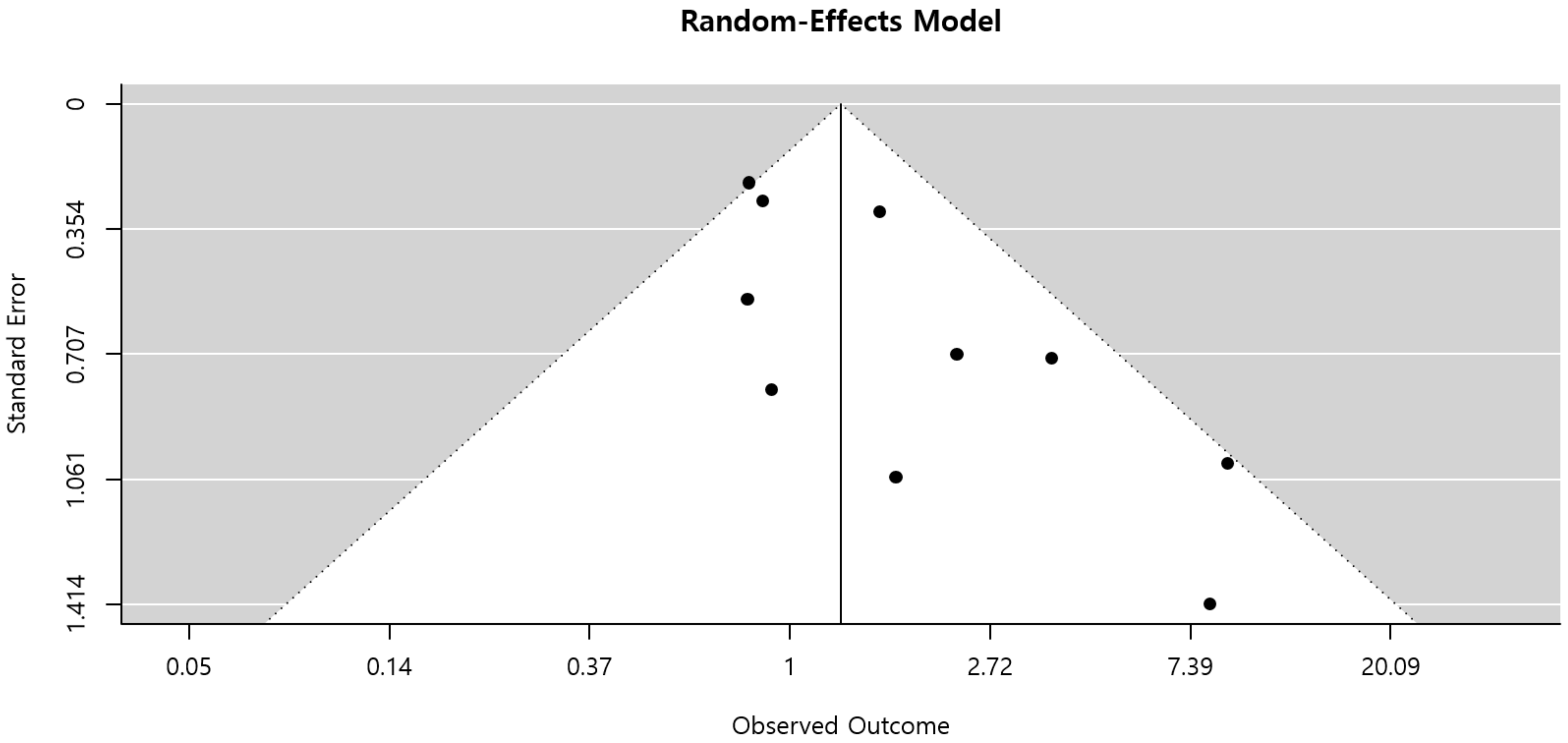

Random-Effects Model
Standard Error
0
0.354
0.707
1.061
1.414
0.05
0.14
0.37
1
2.72
7.39
20.09
Observed Outcome
